# Osseous hemangioma of the seventh cervical vertebra with osteoid formation mimicking metastasis: a case report

**DOI:** 10.1186/1752-1947-3-92

**Published:** 2009-11-02

**Authors:** Stefan Lakemeier, Christina Carolin Westhoff, Susanne Fuchs-Winkelmann, Markus Dietmar Schofer

**Affiliations:** 1 Department of Orthopaedics, University Hospital Giessen and Marburg, Location Marburg, Baldingerstrasse, 35033 Marburg, Germany; 2 Institute of Pathology, University Hospital Giessen and Marburg, Location Marburg, Baldingerstrasse, 35033 Marburg, Germany

## Abstract

**Introduction:**

We report the case of an osseous hemangioma located in the seventh cervical vertebra with reactive osteoid formation and non-typical findings in the radiological and the histopathological examination, mimicking metastasis of a malignant tumor. To our knowledge, this is the first description of such a case in the literature.

**Case presentation:**

A 44-year-old otherwise healthy Caucasian German woman presented with a discrete sensorimotor loss of both upper limbs. Radiologically, an osteolysis in the seventh cervical vertebra suggestive of metastasis of a malignant neoplasm was diagnosed. After performing corporectomy and cage implantation of C7 on the patient, the histopathological examination was complicated by marked osteoid formation obscuring the true diagnosis of an osseous hemangioma with reactive osteoid formation.

**Conclusion:**

Though hemangioma of the bone is a rare tumorous lesion in the cervical spine, it has to be taken into consideration as a reason for neck pain and sensomotoric loss of the upper limbs. Atypical radiological and histopathological presentations may hinder determination of the correct diagnosis. The treatment of such lesions must follow clinical guidelines but may be difficult to define in some cases when the correct diagnosis is not known at the time when therapy starts.

## Introduction

Hemangioma is one of the most common benign tumors of the spine with a reported prevalence of 10% to 12% in the general population [1]. The vast majority of patients with vertebral hemangioma stay asymptomatic. Occasionally, in about 1% of cases, vertebral hemangiomas become symptomatic causing neural arch expansion, vertebral body enlargement or direct compression of the thecal sac or nerve roots [2,3]. Vertebral hemangiomas develop most frequently in the thoracic spine followed by the lumbar spine [4,5]. Cervical lesions are rare. Laredo *et al*. could only find cervical lesions in 7% of their series. Vertebral hemangiomas are often discovered incidentally on screening radiographs or computed tomography (CT) scans [6].

Typically, osseous hemangiomas appear radiographically as lytic medullary lesions with thickened vertical striations, resembling a "corduroy cloth" on X-ray examination or a polka-dot pattern in CT scan cross section [7]. Histologically, osseous hemangiomas display cavernous or capillary blood vessels of mostly mature appearance.

This is a rare case of a seventh cervical vertebra affected by an unusual histological type of hemangioma of the bone with reactive osteoid formation with neither typical radiological nor histopathological findings.

## Case presentation

**Figure 1:**
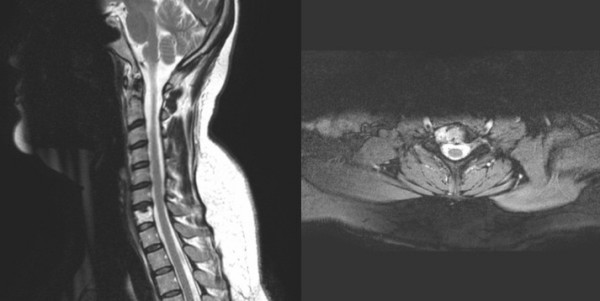
**Axial and sagittal T2-weighted MRI images of cervical spine**. Osteolysis of C7, compromising the stability of the vertebra.

We present the case of a 44-year-old, otherwise healthy, Caucasian German woman who had been experiencing paresthesia in both forearms and hands for 3 months. Occasionally, she had pain and paresthesia in her face when moving her head. Physical examination showed normal muscle strength in both upper limbs and a discrete sensory loss. Movement of the cervical spine was almost free but painful at the end of the motion range. X-ray showed osteolysis of C7. Magnetic resonance images (MRI) indicated increased signal intensity on T2-weightened images. The osteolysis was diagnosed as a metastatic osteolysis of C7 (Figure 1). The CT scan showed that the stability of the vertebra was compromised. "Hemangioma-typical" radiological findings could not be observed either on MRI or CT scans. Angiography did not show an arteriovenous malformation around the lesion and no accumulation of contrast medium was found in the vertebra. The laboratory investigations, including blood count, electrolytes, renal and liver values and infection parameters, were normal.

A complete staging including thoracic and abdominal CT scans, skeleton scintigraphy and MRI of the whole spine revealed no further lesions. The osteolysis was regarded as an unstable metastasis from an unknown primary tumour. Therefore we decided to perform corporectomy of the seventh cervical vertebra and implantation of a customary titanium cage via a lateral approach. A biopsy was ruled out because the lesion was already seen as unstable. We did not want to risk a pathologic fracture with further complications for the patient.

During corporectomy, an intra-operative consultation was performed. The frozen sections showed a cellular lesion displaying spindle-shaped cells with predominantly small, partly elongated nuclei with moderate chromatin density interspersed with immature, partly calcified osteoid. Thus, the intra-operative diagnosis was a mesenchymal tumor.

The postoperative recovery was uneventful and the patient's complaint of neck pain and paresthesia in the forearms disappeared soon. Ten days after surgery she was discharged.

**Figure 2:**
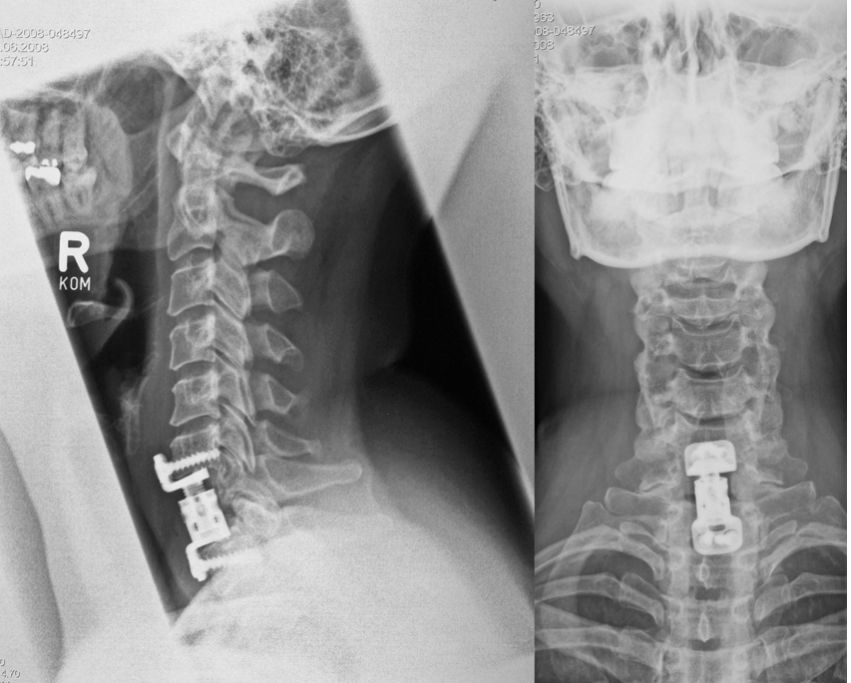
**Radiography of the cervical spine in two levels**. Six months after corporectomy of C7 and cage implantation which show that the correct position of the cage has been maintained and the physiological alignment of the cervical spine is affirmed.

Further microscopic examination of the paraffin-embedded tissue revealed vessel-like structures adjacent to and within the dense mesenchymal cells as well as osteoid (Figure 2). Typical linings of osteoblasts or osteoclastic giant cells were not observed. Thus, our working diagnosis was that of a mesenchymal lesion consistent with an aneurysmal bone cyst, in particular with a solid variant because of the solid areas containing capillaries and osteoid.

This atypical presentation represented a severe diagnostic challenge prompting consultation with a specialist pathologist. Intensive immunohistochemical work-up by the pathologist revealed a distinct vascularization of the lesion with partly collapsed sinusoidal and slit-like blood vessels (Table 1). Criteria of malignancy such as cytologic atypia or an increased mitotic rate were not fulfilled; the lesion was classified asa hemangioma with reactive osteoid formation.

**Table 1:** Overview of immunohistochemical reaction pattern in the lesion

Immunohistochemical marker	Staining pattern
CD31, CD34	Endothelial cells positive
Vimentin, Actin	Endothelial cells, fibroblasts and prae-osteoblasts positive
Desmin, S100	Endothelial cells negative
Ki 67	Less than 1% of nuclei

**Figure 3:**
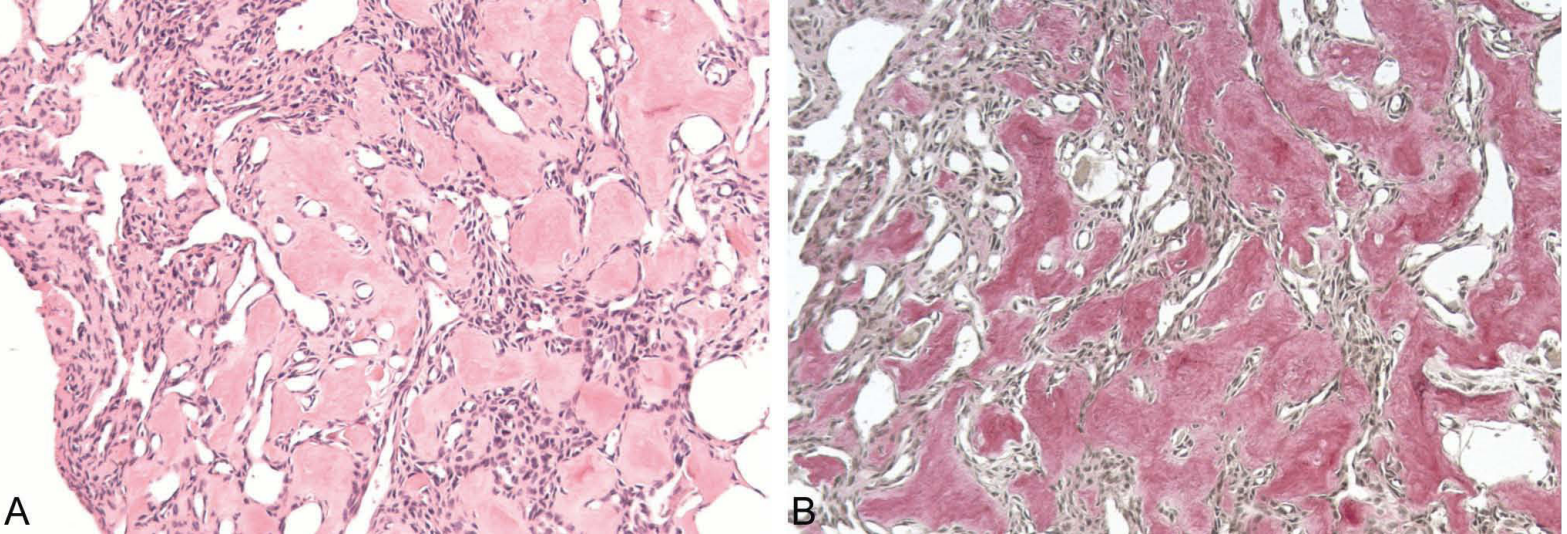
**Histological aspect of the paraffin-embedded material received for intraoperative examination**. Partly calcifying osteoid is the most striking feature, interspersed are partly sinusoidal, partly slit-like, blood vessels as well as more densely arranged areas with spindle-shaped to elongated mesenchymal cells.

Since the final histopathological diagnosis rendered was "hemangioma with reactive osteoid formation", we recommended postoperative radiotherapy, which was refused by the patient. Six months later movement of the cervical spine was painless with a loss of rotation of 20 degrees. There was no sensorimotor loss in the upper limbs. X-ray examination affirmed a good and maintained position of the cage and a normal alignment of the cervical spine (Figure 3).

## Discussion

Vertebral hemangioma is a relatively frequent benign lesion that accounts for a small percentage of surgical biopsies or resections. The radiographic appearance is usually quite characteristic with lytic medullary lesions displaying vertical striations, which appear as "polka-dot" pattern in CT cross sections [7]. Three histologically different types of hemangioma are differentiated: the cavernous, the capillary and the mixed type, displaying usually well-differentiated blood vessels of different sizes [7]. Cavernous hemangiomas are characterized by large, closely clustered dilated blood vessels, not separated by normal bone tissue. Contrarily, capillary hemangioma display thin-walled capillaries of different sizes, separated by normal bone tissue or stroma [8]. Cavernous hemangiomas are most common, followed by the mixed type. Capillary hemangiomas make up the minority [7].

In our case, the predominant histologic pattern of the patient displayed spindle-shaped cells with interspersed, immature and partly calcified osteoid. Further microscopic examination of the paraffin-embedded tissue revealed vessel-like structures adjacent to and within the dense mesenchymal cells. These partly sinusoidal, partly slit-like, blood vessels are indicative of the capillary type of hemangioma. However, the proper diagnosis was obscured by a marked osteoid formation between the vessels, which has been described only on rare occasions [9], and was not congruent with the typical histological appearance of a hemangioma as described above.

Also, the typical radiographic pattern of a vertebral hemangioma, with coarse vertical striations and/or a "polka-dot" pattern on MRI and "honeycomb appearance" on plain films, rather characterizes the cavernous type which may account for the pre-operative diagnostic difficulties and the suspicion of a metastastatic osteolysis of C7. Robin *et al*. found out that the most valuable neuroimaging factor for predicting capillary type are large soft tissue appearances and resorption of bone on CT scans [10]. The signal on T1-weightened MRI is normally isodense but increased on a T2-weightened MRI. Pastushyn *et al*. describe that in the case of "mixed hemangiomas", radiological findings are often untypical and the diagnosis can not be obtained before operation [8]. We postulate that the reactive osteoid formations are responsible for the non-typical radiological findings. Our diagnostic and therapeutic course is along current clinical guidelines described in the literature both for hemangiomas involving the vertebral body and newly occurred vertebral metastases of unknown origin compromising vertebral stability [8,9,11].

## Conclusion

Although hemangiomas of the cervical spine are rare, they have to be considered as a reason for severe neck pain with associated sensorimotor loss of the upper limbs. Vertebral hemangiomas of bone can compromise vertebral stability and lead to pathological fracture with severe consequences for the patient. This case shows that vertebral hemangiomas can mimic vertebral osteolytic metastasis with atypical radiological and histopathological findings. The diagnostic course to reach the correct diagnosis can be complex. We used diverse expensive and time-consuming radiological imaging modalities and histopathological examinations, and emphasise that they must be performed along clinical guidelines to prevent a wrong diagnosis and possibly wrong and dangerous treatment.

## Abbreviations

CD: cluster of differentiation; CT: computed tomography; Ki 67: antibody Ki-67; MRI: magnetic resonance image

## Consent

Written informed consent was obtained from the patient for publication of this case report and any accompanying images. A copy of the written consent is available for review by the editor-in-chief of this journal.

## Competing interests

The authors declare that they have no competing interests.

## Authors' contributions

LS, WCC, FWS and SMD analyzed and interpreted the patient data regarding the osseous hemangioma. SMD carried out the surgical intervention on the patient, WC performed the histopathological examinations. LS was the main author of the manuscript. All authors read and approved the final manuscript.
